# Reversibly Tuning Electrochemiluminescence with Stimulated Emission Route for Single-Cell Imaging

**DOI:** 10.34133/research.0257

**Published:** 2023-10-18

**Authors:** Cheng Ma, Xiaodan Gou, Zejing Xing, Min-Xuan Wang, Wenlei Zhu, Qin Xu, Dechen Jiang, Jun-Jie Zhu

**Affiliations:** ^1^ School of Chemistry and Chemical Engineering, Yangzhou University, Yangzhou 225002, P. R. China.; ^2^ State Key Laboratory of Analytical Chemistry for Life Science, State Key Laboratory of Pollution Control and Resource Reuse, School of Chemistry and Chemical Engineering, School of the Environment, Nanjing University, Nanjing 210023, P. R. China.

## Abstract

Electrochemiluminescence (ECL) has established itself as an excellent transduction technique in biosensing and light-emitting device, while conventional ECL mechanism depending on spontaneous emission of luminophores lacks reversibility and tunable emission characters, limiting the universality of ECL technique in the fields of fundamental research and clinical applications. Here, we report the first observation of stimulated emission route in ECL and thus establish a reversible tuning ECL microscopy for single-cell imaging. This microscopy uses a focused red-shifted beam to transfer spontaneous ECL into stimulated ECL, which enables selective and reversible tuning of ECL emission from homogeneous solution, single particles, and single cells. After excluding other possible competitive routes, the stimulated ECL emission route is confirmed by a dual-objective system in which the suppressed spontaneous ECL is accompanied by the enhanced stimulated ECL. By incorporating a commercial donut-shaped beam, the sharpness of single-cell matrix adhesion is improved 2 to 3 times compared with the counterpart in confocal ECL mode. The successful establishment of this stimulated emission ECL will greatly advance the development of light-emitting device and super-resolution ECL microscopy.

## Introduction

The profound transformation of cellular and molecular biology in recent decades has coincided with advances in new analytical technologies [1]. Because of cellular diversity and interindividual variation, single-cell analysis strategies offer exciting opportunities, alongside challenges, for investigating real biomolecular interactions in single cells without significant batch effects [2,3]. Among a multitude of single-cell analysis methods, imaging techniques have proven promising and very useful due to their high throughput and spatiotemporal resolution [4,5]. Accordingly, various microscopes depending on diversified electrochemical or spectroscopic principles have been developed for an in-depth understanding of single-cell dynamics from different aspects [6].

Electrochemiluminescence (ECL) is a special luminescence form that is excited by the chemical reactions among electrogenerated radicals and then relaxes the energy by emitting a photon [7]. Therefore, the input electrochemical signals are well separated from the output light signals. Virtually no excitation light and photobleaching disturb the analytical measurements, greatly enhancing the sensitivity and stability [8–11]. Integrating these merits with the spatiotemporal resolution of optical microscopy, ECL microscopy has recently discovered many interesting phenomena such as the chemical lens effect, single particle blinking, ECL waveguide, and C–N dissociative coreactant mechanism [12–15]. In application domains, various single-particle catalysis and single-cell analysis based on ECL microscopy have supplied the candidate strategies in chemical imaging toolboxes. These works involved imaging cellular membranes, intracellular biomolecules, organelles, cell adhesion, and so on [16–25]. The reconstruction techniques and signal amplification in ECL microscopy further realized single-molecule sensitivity and the super-resolution level [26–28]. Therefore, it is important to introduce new concepts into ECL realm because these discoveries may transform the ECL scenario and promote the hitherto single-cell microscopy. Given that many ECL imaging methods lack reversibility to tune ECL such as ECL loss in photobleaching and ECL burst by oxygen doping, it is necessary to design a reversibly responsive ECL imaging switch, which implied a good stability and long-term recycle of ECL technique.

Though ECL microscopy has been applied in many fields, insights into the mechanisms underlying ECL processes are still rigorous foundation for the applications of ECL microscopy [15,29–34]. Many works have recently focused on the regulation of ECL emission state via new approaches, such as adopting surface state emission in nano-emitters [35] or ECL resonance energy transfer (ERET) systems [36]. For nano-emitters, surface engineering controls the energy levels and defect sites on surface. Because of the surface-sensitive nature, ECL intensity and wavelength can be regulated by various physical and chemical treatments like surface doping, passivation, corrosion, and ligand exchange [37–39]. Compared with the band-gap transition in the photoluminescence (PL) process, ECL often shows a red-shifted spectrum from surface state with lower energy [35]. Alternatively, ERET uses a nonradiative energy transfer mechanism from an excited state of ECL emitters to a proximal energy acceptors through long-range dipole–dipole interactions. As a result, the ECL emission can be enhanced or quenched or changed in wavelength, determined by the extent of spectral overlap and the distance between donor and acceptor. The ERET phenomenon further facilitates the developments of new ECL sensors mostly based on the interaction between energy transfer efficiency and analytes [36,40]. Additionally, ECL emission state can be regulated by other approaches such as aggregation-induced enhancement (AIE) [41,42], crystallization-induced emission (CIE) [43], photoinduced upconversion [44,45], hydrogen bonding [46], functional group modification [47], and thermally activated delayed fluorescence [48]. Nevertheless, the ECL emission state in these regulation strategies follows spontaneous emission route according to Boltzmann distribution. Thus, further efforts are required to investigate other ECL emission routes, which pave the way for advanced ECL analysis and imaging techniques.

**Fig. 1.**
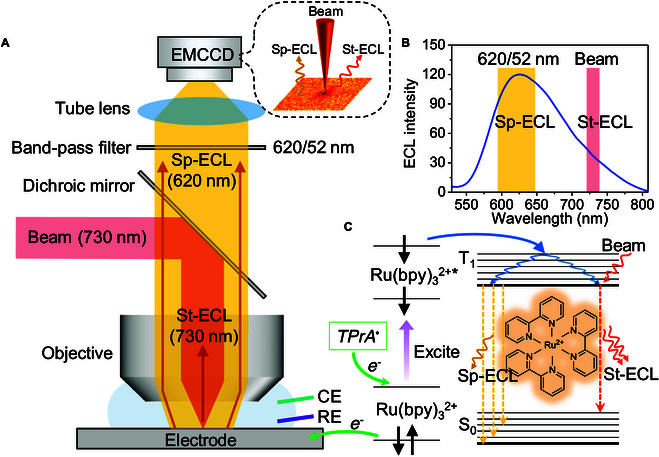
(A) Schematic illustration of ECL microscopy with a red-shifted beam. Spontaneous ECL (Sp-ECL; λ_max_ = 620 nm) is generated when a voltage is applied in the electrolyte containing Ru(bpy)_3_^2+^ and coreactant TPrA. A red-shifted beam (730 nm) transfers Sp-ECL (λ_max_ = 620 nm) into stimulated ECL (St-ECL, 730 nm), which is then blocked by band-pass filter (620/52 nm) and forms a dark spot in EMCCD. (B) ECL spectrum of Ru(bpy)_3_^2+^ with the wavelength of maximum emission (λ_max_) at 620 nm. Sp-ECL is collected in 620/52-nm spectral channel. A red-shifted beam (730 nm) lies in the red tail of ECL spectrum to induce St-ECL. (C) Excitation and emission steps of Sp-ECL and St-ECL. The electron transfer reactions facilitate the establishment of triplet excited states (T_1_) from ground state (S_0_). After intersystem crossing and vibration relaxation to the lowest triplet state (T_1_) energy level, excited state Ru(bpy)_3_^2+^* generates either Sp-ECL emission (λ_max_ = 620 nm) or St-ECL emission (730 nm) induced by beam via stimulated emission route.

Here, we discover the stimulated emission route of ECL and thus establish a reversible tuning ECL microscopy with a red-shifted beam for single-cell imaging (Fig. 1). Both spontaneous and stimulated emission routes are a transition form of released energy for excited state [49]. The difference is that spontaneous emission happens without the presence of an external stimulus field, and emitted photons are random in direction and wavelength. In stimulated emission, however, electron on excited state interacting with an incident electromagnetic wave drops to a lower energy level and emits a new photon that has the same direction and wavelength as the incident wave. The stimulated emission theory was proposed by Einstein and subsequently became the fundamentals of LASER industry and STED microscopy [50–52]. However, the question as to whether stimulated emission route exists in ECL realm has remained unaddressed. Here, we observed ECL energy transfer of Ru(bpy)_3_^2+^ by stimulated emission. Usually, Ru(bpy)_3_^2+^ can generate an orange-red ECL (~620 nm) after reacting with coreactant tri-*n*-propylamine (TPrA). But under the irradiation of external 730-nm beam, the ECL emission wavelength shift from 620 to 730 nm, causing an opposite trend in 620- and 730-nm channels. The stimulated ECL (St-ECL) route is further validated after excluding competitive routes such as photobleaching, photocorrosion, and photothermal effects. The universality of St-ECL emission was tested at homogeneous solution, single particles, and single cells whose ECL intensity is selectively and reversibly switched off and on by the red-shift beam. The stimulated emission route of ECL was validated at both “oxidative-reduction” and “catalytic” routes. Finally, the ECL sharpness at cell contour is improved 2 to 3 times with the aid of donut red-shift beam because of the stimulated emission route.

## Results

### Construction of stimulated emission ECL microscopy

To investigate whether the St-ECL route truly exists, we first need to introduce a red-shifted beam into an upright ECL microscope (Fig. 1A). The beam wavelength is essentially determined by the photophysical properties of ECL luminophores. Here, the widely used Ru(bpy)_3_^2+^ is chosen as the model ECL system due to its high ECL efficiency and stability. As shown in Fig. S1, Ru(bpy)_3_^2+^ can generate an orange-red spontaneous ECL (Sp-ECL; ~620 nm) with the aid of coreactant TPrA through the classic “oxidative-reduction” mechanism (Eqs. 1 to 4) and other possible mechanisms [53]. Therefore, we chose a 730-nm beam, located at the red edge of ECL spectrum of Ru(bpy)_3_^2+^ (Fig. 1B), for 2 reasons. First, the 730-nm beam can induce stimulated emission route of excited state Ru(bpy)_3_^2+^* molecules according to spectroscopic rationale (Fig. 1C and Fig. S2) [54,55]. Second, 730-nm photon has a low energy so as not to excite Ru(bpy)_3_^2+^ PL (Fig. S3) [56].

$$\mathrm{Ru}{\left(\mathrm{bpy}\right)}_{3}^{2+}-e^{-}⟶\mathrm{Ru}{\left(\mathrm{bpy}\right)}_{3}^{3+}$$ (1)

$$\text{TPrA}-e^{-}\to {\text{TPrA}}^{+•}⟶{\text{TPrA}}^{•}+H^{+}$$ (2)

$$\mathrm{Ru}{\left(\mathrm{bpy}\right)}_{3}^{3+}+{\text{TPrA}}^{•}⟶\mathrm{Ru}{\left(\mathrm{bpy}\right)}_{3}^{2+*}+P1$$ (3)

$$\mathrm{Ru}{\left(\mathrm{bpy}\right)}_{3}^{2+*}⟶\mathrm{Ru}{\left(\mathrm{bpy}\right)}_{3}^{2+}+\mathrm{hv}\left(\text{Sp-ECL}\right)$$ (4)

or

$$\mathrm{Ru}{\left(\mathrm{bpy}\right)}_{3}^{2+*}+\text{Beam}⟶\mathrm{Ru}{\left(\mathrm{bpy}\right)}_{3}^{2+}+\mathrm{hv}\left(\mathrm{St-}\mathrm{ECL}\right)$$ (5)

**Fig. 2.**
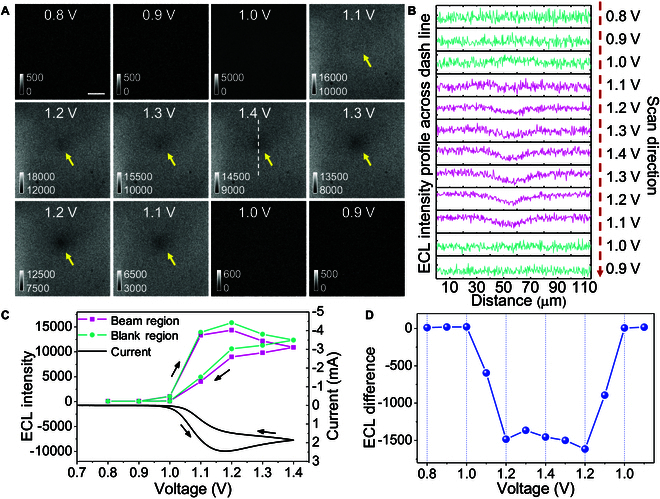
(A) ECL snapshots during a potential cycle (0.8 to 1.4 V) at a scan rate of 0.1 V/s in 200 mM phosphate-buffered saline (PBS) (pH 7.0) containing 200 μM Ru(bpy)_3_^2+^ and 20 mM TPrA. The arrow indicates the St-ECL region irradiated by the beam. Exposure time is 200 ms. The focused beam power is 8.1 μW/μm^2^. Scale bar (white) is 30 μm. (B) ECL intensity profiles across the dashed line in (A) during the potential cycle. (C) ECL intensity in beam irradiation and blank regions in (A) as a function of electrode potential and the corresponding cyclic voltammetry curve. (D) Quenching degree of Sp-ECL by the beam irradiation as the potential is cyclically scanned from 0.8 to 1.4 V.

Subsequently, the collimating 730-nm beam flooded into the back focal plane of objective and produced a focused spot on electrode surface (Fig. 1A). The point spread function (PSF) of the focused spot featured a full width at half maximum (FWHM) of 3.2 μm in the lateral direction (Fig. S4). To prevent the back-propagating 730-nm beam from entering the camera and disturbing the record of the ECL signal, a premium bandpass filter (center wavelength 620 nm, FWHM 52 nm) was placed in front of the camera so that only Sp-ECL emission was collected (Fig. 1A and Fig. S4). Then, we acquired the ECL image sequence with a cyclic voltammetry sweeping in freely diffusing Ru(bpy)_3_^2+^ and TPrA homogeneous solution (Fig. 2). As the potential scanned beyond 1.0 V, both the current and overall ECL intensity started to increase due to the “oxidative-reduction” route (Eqs. 1 to 4) and other coexisted ECL routes [15,53]. However, the region irradiated by the beam showed a relatively dark Sp-ECL compared with other blank regions. The ECL difference between the 2 regions became more obvious as the overall ECL intensity increased with cyclic voltammetry scanning (Fig. 2). The ECL difference in the reverse scan is larger than that in the forward scan because the ECL layer in the reverse scan is relatively thinner due to the electrochemical reduction of Ru(bpy)_3_^3+^ molecules as well as TPrA^+•^. To prove this point, we compared the cell pattern in the forward and reverse scan. The cell patterns in the forward scan showed narrow bright edges at cell–cell junctions because the thickness of ECL layer increases to several micrometers (Fig. S5). In contrast, these bright edges at cell–cell junctions are not so clear when the potential is scanned in the reverse direction. It indicated that the thickness of ECL layer became thinner in this situation. Because St-ECL depended on the interaction between focused red-shift beam and ECL layer, the efficiency of St-ECL became lower if the ECL layer became thicker beyond the electrode surface, out of the focused beam. In contrast, when the beam was switched off, Sp-ECL turned into uniform distribution on electrode surface during the potential sweeping (Figs. S6 and S7 and Movie S1). Because St-ECL is a competitive emission process with Sp-ECL for excited state Ru(bpy)_3_^2+^* (Eqs. 4 and 5), the ECL difference suggests that the 730-nm beam may induce St-ECL, which inevitably decreases the probability of Sp-ECL and causes the dark spot.

### Reversibility of stimulated emission ECL

To test the reversibility of suppressed Sp-ECL induced by the beam, we continuously switched the beam on and off under a constant 1.3-V potential (Fig. S8 and Movie S2). When the beam turned on, Sp-ECL in the irradiation region instantly dropped down to a low level but then rebounded immediately to the previous level once the beam turned off. The good reversibility indicates that the suppressed Sp-ECL is not attributed to the irreversible photocorrosion of indium tin oxide (ITO) electrode. To test the photothermal effect of the beam on ITO electrode, we measured the absorption spectrum of ITO electrode (Fig. S9). The negligible absorption of ITO at 730 nm indicates very little photothermal effect. In addition, according to Arrhenius equation, photothermal effect should cause an enhanced Sp-ECL rather than a suppressed Sp-ECL [24,25]. In stimulated emission route, the probability of spontaneous emission inversely correlates with the number of incident beam photons [57]. Accordingly, we found that increasing beam power intensified the quenching degree of Sp-ECL (Fig. S10). Note that the overall quenching degree is not so high because Ru(bpy)_3_^2+^ is freely diffusing in homogeneous solution.

**Fig. 3.**
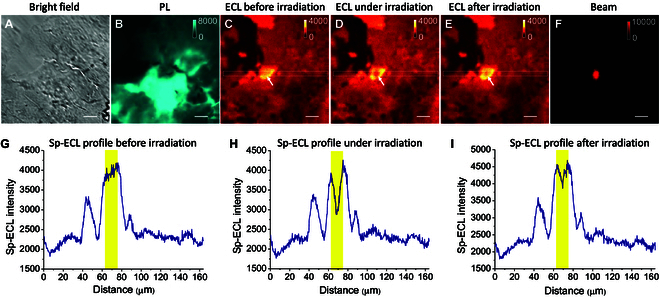
Bright field (A) and PL (B) images showing Nafion polymer film on ITO electrode. Ru(bpy)_3_^2+^ molecules are loaded into the Nafion film because PL image shows the PL emission of Ru(bpy)_3_^2+^ (~620 nm) in the Nafion film under 460-nm light excitation. ECL images before (C), under (D), and after (E) 730-nm beam irradiation. (F) The 730-nm beam spot after removing the band-pass filter (620/52 nm). Scale bar (white) is 20 μm. Electrolyte is 200 mM PBS (pH 7.0) containing 100 mM TPrA. Constant potential: 1.3 V. (G to I) Sp-ECL intensity lateral profiles across the dashed boxes in (C) to (E). The yellow zones show the Sp-ECL profiles across the beam irradiation region in (C) to (E), which is marked with the arrows.

Under the beam irradiation, photobleaching is another possible competitive route. So we used Nafion film to immobilize Ru(bpy)_3_^2+^ on ITO surface [58]. If the quenching of Sp-ECL is totally caused by photobleaching, Ru(bpy)_3_^2+^ immobilized in Nafion film will be irreversibly decomposed and the irradiation region will retain dark Sp-ECL after the beam is switched off [59]. As shown in Fig. 3, Ru(bpy)_3_^2+^ immobilized in Nafion film shows bright Sp-ECL under 1.3-V potential with TPrA as coreactant. The difference between PL and ECL images is attributed that ECL depends on not only the doping amount but also the electrochemical reaction rates of Ru(bpy)_3_^2+^ and TPrA. When the beam turns on, Sp-ECL at irradiation region is quenched to 72% of the previous level. Thus, we observe a valley at Sp-ECL profile (highlights in Fig. 3H). However, Sp-ECL in the irradiation region immediately recovered to a great extent (92%) when the beam turned off (Fig. 3I). Although Sp-ECL did not fully recover to the previous level probably due to photobleaching, this result demonstrates that the suppressed Sp-ECL is not primarily caused by the photobleaching effect.

For Ru(bpy)_3_^2+^, ECL and PL should obey the same emission pathway despite the different excitation pathways (Figs. S1 and S2). We also tested whether spontaneous PL (Sp-PL) of Ru(bpy)_3_^2+^ can be reversibly tuned by the 730-nm beam. As shown in Fig. S11, we introduced another 405-nm beam as excitation light of Ru(bpy)_3_^2+^, which was focused on the back focus plane of objective and formed a collimated excitation light for wide-field PL imaging. After Ru(bpy)_3_^2+^-doped silica nanoparticles (RuDSNs) were modified on electrode, we observed many RuDSNs emitting Sp-PL (λ_max_ = 620 nm) under 405-nm excitation. Meanwhile, another 730-nm beam was aligned to focus on a single RuDSN to induce stimulated emission. The irradiated single RuDSN showed a continuous switching Sp-PL states between quenching and recovery as the 730-nm beam was switched ON and OFF, respectively. On the contrary, other RuDSNs without 730-nm irradiation showed only a continuous PL decay due to the photobleaching effect under 405-nm excitation (Fig. S12). These results suggest that the 730-nm beam probably transfers Sp-PL at 620 nm into stimulated emission at 730 nm, which is consistent with St-ECL mechanism and previous reports [54,56,60]. However, the processes involved in St-ECL are more complex than stimulated fluorescence. First, the excitation of ECL depended on the high-energy electron-transfer reactions to form excited state rather than the direct absorption of external photons. Therefore, the electrochemical workstation or potentiostat is necessary to offer voltage that triggers St-ECL. Second, the ECL quantum efficiency is much lower than the fluorescence quantum efficiency because ECL intensity relied on the various chemical reaction rates between coreactant and luminophore. For the classic luminophore Ru(bpy)_3_^2+^, the ECL quantum efficiency is no more than 5%. Therefore, the St-ECL quenching is not as significant as the stimulated fluorescence depletion given the weak ECL intensity. Third, because of the free diffusion of luminophore and coreactant, the excited luminophore will diffuse some distance before emitting ECL. It means that the position of ECL photon is not exactly the same as the reaction position where luminophore is excited. Compared with stimulated fluorescence, St-ECL depends not only on photophysical process but also on heterogeneous and homogeneous (electro)chemical reactions.

### ECL energy transfer from Sp-ECL to St-ECL

**Fig. 4.**
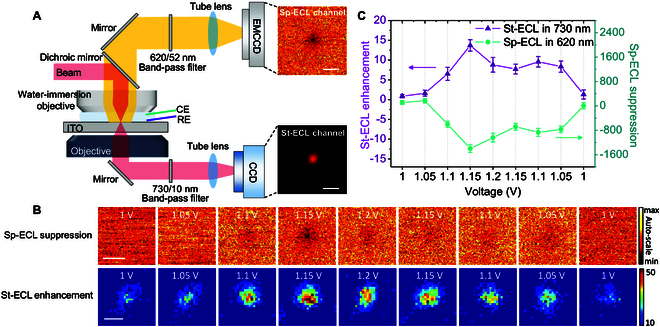
(A) Schematic illustration of a coaxial dual-opposing-objective system to simultaneously record the reflected Sp-ECL (620-nm channel) and the transmitted St-ECL (730-nm channel). Scale bar (white) is 10 μm. (B) Synchronous Sp-ECL and St-ECL signals in the beam irradiation region during a CV scan from 1 to 1.2 V. The first row is the reflected Sp-ECL images from upright microscope where the dark spot is the irradiation region. Scale bar (white) is 10 μm. The second row is the transmitted St-ECL from inverted microscope after beam background is deducted. Scale bar (white) is 5 μm. Scan rate is 0.1 V/s. Electrolyte is 200 mM PBS (pH 7.0) containing 400 μM Ru(bpy)_3_^2+^ and 30 mM TPrA. Exposure time of EMCCD and CCD is 200 and 100 ms, respectively. (C) Profiles of St-ECL enhancement and Sp-ECL suppression with voltage sweeping. Error bars indicate the standard deviation (*n* = 3). The data of St-ECL enhancement and Sp-ECL suppression are extracted from (B).

To confirm the transformation from Sp-ECL to St-ECL under 730-nm beam irradiation, we built a coaxial dual-opposing-objective system to simultaneously record the reflected 620-nm Sp-ECL and the transmitted 730-nm St-ECL (Fig. 4). First, the collimating 730-nm beam flooded into the back focal plane of the upper objective and generated a focused spot on ITO electrode surface. Then, the focused beam passed through the ITO electrode and was collected by the inverted objective. Accordingly, the dual-objective configuration enabled us to simultaneously record Sp-ECL and St-ECL because St-ECL possessed the same wavelength and direction with beam. As shown in Fig. 4B, during a cyclic voltammetry scan, the upper camera showed that Sp-ECL in the irradiation region decreased. Meanwhile, the lower camera showed that 730-nm light intensity increased after subtracting the original beam background (Fig. 4B and Fig. S13). The 620-nm Sp-ECL difference shows an exactly opposite trend of 730-nm difference (Fig. 4C). Because of the competitive relation between Sp-ECL (620 nm) and St-ECL (730 nm), this result confirms the energy transfer by stimulated emission route induced by beam (Eqs. 4 and 5).

According to stimulated emission theory, energy transfer efficiency should depend on the spectral match between beam wavelength and ECL spectrum of luminophore. Thus, we tested ECL energy transfer by using other beam wavelengths and ECL luminophores, respectively. First, a longer wavelength 808-nm beam was used to quench Sp-ECL of Ru(bpy)_3_^2+^ (Fig. S14). Compared with the 730-nm beam, the 808-nm beam shows a less inhibition of Sp-ECL. It is reasonable because the 808-nm beam is located at the far end of ECL spectrum of Ru(bpy)_3_^2+^ and, thus, has less cross-section to produce ECL energy transfer according to spectroscopic rationale [55,61]. Second, a luminol derivative (L012) was used as another luminophore, whose ECL spectrum ranges from 450 to 700 nm (Fig. S15). When the 730-nm beam located beyond the ECL spectrum of L012 was used to quench ECL, the irradiation region, however, showed the same Sp-ECL intensity as the blank region. It suggests that the 730-nm beam does not induce energy transfer of L012 due to the mismatch between beam wavelength and ECL spectrum of L012. However, when a 532-nm beam was used instead of the 730-nm beam, the irradiation spot became dark when the 532-nm beam turned on because the 532-nm beam located at the red edge of ECL spectrum of L012 (Fig. S16). When the 532-nm beam turned off, the ECL intensity at the irradiation spot recovered to the previous value, the same ECL intensity at the blank areas. Therefore, St-ECL only occurred when the wavelength of irradiation beam matched the ECL spectrum of luminophore.

### St-ECL microscopy for imaging single entities

**Fig. 5.**
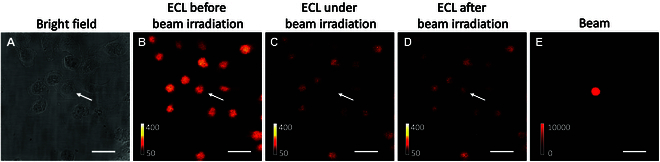
(A) Bright field image of HeLa cells on ITO electrode. (B to D) ECL images of HeLa cells before, under, and after beam irradiation. The arrow indicates the single cell irradiated by the 730-nm beam, which selectively suppresses the single cell, while other cells remain bright. (E) The 730-nm beam spot after removing the band-pass filter (620/52 nm). Cells are fixed with paraformaldehyde for 30 min and pretreated with 0.1% Triton X-100 for 10 min. Voltage: a constant 1.3 V (versus Ag/AgCl). Electrolyte: 200 mM PBS (pH 7.0) containing 2 mM Ru(bpy)_3_^2+^. Scale bar (white) is 30 μm.

After the confirmation of ECL energy transfer by stimulated emission route, the beam was used to selectively and reversibly modulate ECL signals from single entities. We synthesized a nano-coreactant by encapsulating aminated carbon dots into hollow silica nanosphere (NCDs@HSN) (Fig. S17). The amine-rich NCDs@HSN significantly improves Sp-ECL of Ru(bpy)_3_^2+^ via catalytic route [62]. When the 730-nm beam was focused on single NCDs@HSN aggregate, Sp-ECL from NCDs@HSN was instantly suppressed, but subsequently recovered after the 730-nm beam turned off. The Sp-ECL lateral profile of NCDs@HSN is exactly complementary to the beam lateral profile, suggesting the positive correlation between ECL quenching degree and the power of beam. A similar result was observed when single NCDs@HSNs were replaced by single cells (Fig. 5). Because intracellular biomolecules with amine moieties serve as coreactants of Ru(bpy)_3_^2+^, intracellular structures, especially cell nuclei, show strong Sp-ECL through catalytic route with Ru(bpy)_3_^2+^ [19]. The colocalization of fluorescence stain of cell nuclei and ECL demonstrated that cell nuclei showed strong ECL signal compared with other compartment in cell (Fig. S18). As shown in Fig. S19A, the cyclic voltammetry curve of Ru(bpy)_3_^2+^ in the ITO electrode modified with attached cells showed a symmetrical oxidation and reduction peak of Ru(bpy)_3_^2+^/Ru(bpy)_3_^3+^. The oxidation and reduction peaks lied in 1.18 and 1.01 V, respectively. In Fig. S19B, the ECL-potential curve showed a maximal ECL intensity around 1.3 V in the ITO electrode modified with attached cells. Therefore, we chose 1.3 V because the ECL intensity reached its peak at this potential when catalytic route is the dominant ECL route with attached cells as coreactants. But when the beam was directed to a single cell (Fig. 5), Sp-ECL from the cell was suppressed while the other cells remained bright. After the beam turned off, Sp-ECL from the cell appeared again, showing a similar ECL intensity as other non-irradiation cells. Note that the ECL intensity of all cells shows a gradually decline because intracellular bio-coreactants are gradually consumed during ECL reactions. Nevertheless, these results demonstrate that stimulated emission route allows to selectively and reversibly modulate ECL signals of single entities through energy transfer from Sp-ECL to St-ECL states.

**Fig. 6.**
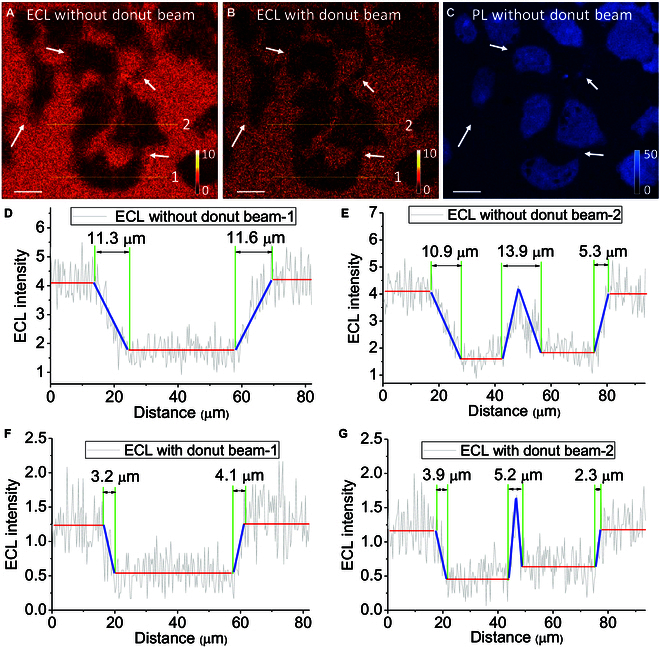
(A) Point-by-point scanning ECL image of cell matrix adhesions when switching off the excitation and donut beams. ECL was generated under a constant 1.4-V potential. Electrolyte is 200 mM PBS (pH 7.0) containing 1 mM Ru(bpy)_3_^2+^ and 100 mM TPrA. (B) ECL imaging under the same condition except for a synchronous donut beam also point-scanning the sample. ECL emission is collected between 550 and 720 nm. (C) PL image of HeLa cell in the same region as in (A) and (B). PL image is obtained by a 470-nm light excitation after cells are stained with Ru(bpy)_3_^2+^ through electrostatic interaction. PL emission is collected between 550 and 680 nm. The arrows indicate the positions of cell adhesion structures like podosomes, lamellae, and ruffles. Cells are fixed with paraformaldehyde for 30 min. Scale bar (white) is 20 μm. (D and E) ECL intensity profiles across the 2 yellow lines (marked by 1 and 2) in (A). (F and G) ECL intensity profiles across the 2 yellow lines (marked by 1 and 2) in (B). The blue lines in (D) and (F) and (E) and (G) compare the resolution of cellular edges.

Because beam pattern determines the shape of St-ECL region, we introduced a donut-pattern beam in a commercial Leica TCS SP8 STED microscope, which enables the donut beam point-by-point scan imaging with galvo mirror systems (Fig. S20). The parameters and pattern of donut beam are shown in Fig. S21. As a result, Sp-ECL in donut center is retained, but Sp-ECL in the periphery is quenched at each site during the beam scan. Reversibly responsive ECL imaging is the foundation of stimulated emission depletion microscopy because the point-by-point scanning by the donut-shaped beam depended on the repeatedly multiple ECL on/off switch of Ru(bpy)_3_^2+^. As shown in Fig. 6A, when we switched off the excitation light and used an electrochemical workstation to generate ECL, cells on ITO electrode show shadow regions because cell-matrix adhesions block the ECL reactions and the diffusion of reagents [21,26,63]. In this case, the oxidative-reduction route is the dominant ECL mechanism in the solution containing 1 mM Ru(bpy)_3_^2+^ and 100 mM TPrA. ITO electrode has more sluggish kinetics for the electrochemical oxidation of TPrA. Thus, to obtain enough ECL signal, the applied voltage is set as 1.4 V rather than 1.3 V because the ECL signal in 1.4 V is larger than the ECL signal in 1.3 V (Fig. S19). The oxidation current was mainly from the irreversible electrochemical oxidation of TPrA. Then, we used a donut beam point-scanning across the same region. Because the beam induced energy transfer through stimulated emission route, the ECL intensity was suppressed under the beam irradiation. However, because the donut-shaped beam retains Sp-ECL from the center, the resolution on identical regions is improved 2 to 3 times by calculating the profiles of transition regions in cellular edges (Fig. 6D to G). Thus, the beam point-scanning across cell matrix adhesions provides an improved spatial resolution of cell contour structures with St-ECL route. This St-ECL imaging is similar to PL imaging mode with stimulated emission route. [54,56,60] Under the PL imaging mode with donut beam, the cell nucleus stained with Ru(bpy)_3_^2+^ shows finer details (Fig. S22), analogous to the result achieved by ECL imaging mode with donut beam.

## Discussion

We discover the stimulated emission route of ECL for the first time and thus establish a reversible tuning ECL microscopy of single cells. For excited state ECL luminophores, a red-shifted beam specifically transfers Sp-ECL into St-ECL according to the spectroscopic rationale. To clarify the reason for ECL energy transfer, various control experiments and a dual-objective system are performed to exclude other possible competitive mechanisms such as photocorrosion, photothermal effect, and photobleaching. The St-ECL emission route is validated in homogeneous solution, single particles, and single cells. In addition, the stimulated emission ECL microscopy enables reversibly and selectively tuning ECL signals from single cells. By using a donut beam to quench Sp-ECL on the periphery, the resolution at cellular edges is improved 2 to 3 times compared with the counterpart in confocal ECL mode. Therefore, we anticipate that the St-ECL energy transfer is a new regulation strategy for ECL switch in addition to ERET and surface state. Stimulated emission ECL route has great potential as a complementary route for designing super-resolution ECL microscopy [26,27]. Also, combining photo-assisted electrochemical devices with St-ECL can open a new avenue to further simplify the setup in St-ECL microscope [64,65]. Because stimulated emission is the principle of modern LASER industry, the reversibility of St-ECL also implied the long-term stability of St-ECL devices involving LASER action. In the field of sensors, if an ECL sensors is supposed to be reused and regenerated in practical applications, reversible regulation is a key point that must be considered when designing the ECL sensors.

## Materials and Methods

### Chemicals

Unless otherwise stated, all the other chemicals and reagents used in this study were of analytical grade quality and were used as received without further purification. Ultrapure water with a resistivity of 18.2 MΩ cm was produced by using a Milli-Q apparatus (Millipore) and used in the preparation of all solutions. Polydimethylsiloxane (PDMS) electrochemical cell was prepared by using Sylgard 184, Dow Corning. Tris(2,2′-bipyridyl)dichlororuthenium(II) hexahydrate [Ru(bpy)_3_^2+^], Nafion, and tripropylamine (TPrA) were purchased from Sigma-Aldrich. Branched polyethyleneimine (PEI; molecular weight = 600) was purchased from Aladdin Reagent Inc. 8-Amino-5-chloro-7-phenylpyrido[3,4-d]-pyridazine-1,4(2H,3H)-dione (L012, a luminol analogue) was bought from Wako Chemical U.S.A. Inc. (Richmond, VA). Triton X-100 was purchased from Macklin Biochemical Co. Ltd. Tetraethyl orthosilicate (TEOS) was purchased from Sinopharm Reagent (Beijing China). Paraformaldehyde solution (4%) was purchased from KeyGEN BioTECH. ITO was purchased from Zhongjingkeyi Technology Co. Ltd. The ITO modified with Ru(bpy)_3_^2+^-doped Nafion membrane was prepared according to the previous method [58]. RuDSNs were synthesized by a water-in-oil microemulsion method [66]. Hollow silica nanosphere (HSN) and aminated carbon dots (NCDs) were synthesized by the Stober method [67] and the classic hydrothermal method [68], respectively. Aminated carbon dots doped hollow silica nanosphere (NCDs@HSN) was prepared by the electrostatic adsorption of NCDs into the pores of the mesoporous HSN. A HeLa cell line was purchased from the Institute of Cell Biology at the Chinese Academy of Sciences (Shanghai, P. R. China) and cultured in Dulbecco's modified Eagle's medium (DMEM) (Life Technologies, Grand Island, NY, USA) containing 10% fetal bovine serum, 100 U/ml penicillin, and 100 μg/ml streptomycin at 37 °C under 5% CO_2_ atmosphere. At the logarithmic growth phase, the cells were transferred at an ITO electrode surface and left undisturbed for 8 h for cell adherence. The smooth surface of ITO electrode allowed HeLa cells to adhere to and grow on it.

### ECL imaging

ECL imaging was performed on a homemade upright configuration microscope [13]. A water immersion objective [Olympus LUMFLN×60, numerical aperture (NA) = 1.10] and an electron multiplying charge-coupled device (EMCCD) camera (Photometrics, Evolve Delta) were used for bright field, PL, and ECL imaging. A potentiostat (CHI 660D electrochemical workstation) was used to control the ITO electrode potential with an Ag/AgCl (saturated KCl) and a Pt wire as the reference electrode and the counter electrode, respectively. The EMCCD was triggered and synchronized by the potentiostat. The 730- and 532-nm laser diodes (HL7302MG, DJ532-40, Thorlabs) were used to induce stimulated emission ECL. A dichroic mirror (FF697-SDi01, Semrock) was used to reflect the 730-nm beam but allowed 620-nm Sp-ECL to pass through. PL imaging was also performed on this microscope by using a 405-nm laser diode (LP405-SF10, Thorlabs) as the excitation light, fluorescent filters, and a dichroic mirror. A coaxial dual-opposing-objective system was established based on a Nikon Ti2-E inverted microscope and an integrating upright microscope with a water immersion objective and EMCCD. Below the water immersion objective is a dry objective (Nikon Plan Fluor ×40, NA = 0.60), which is mounted on the inverted microscope. The ITO electrode is placed between the 2 objectives. A CCD camera (Pike F-032B, Allied Vision) on the inverted microscope was used to collect 730-nm light. Other optical elements and optomechanical components were purchased from Semrock and Thorlabs Inc. Transmission electron micrographs (TEMs) were measured on a JEOL JEM 200CX transmission electron microscope using an accelerating voltage of 200 kV. Ultraviolet–visible (UV–vis) absorption spectra were obtained using a UV-3600 spectrophotometer (Shimadzu). The ECL and PL spectra were collected by a homemade spectral acquiring system [69]. The setup was composed of a monochromator (Acton SP2300i, PI), a spectrograph CCD (PIXIS 400BR_excelon, PI), a grating (grating density: 300 L/mm; blazed wavelength: 500 nm), a CHI 660D electrochemical workstation, a cuvette holder, and a light-tight cover. Cellular fluorescence imaging and cell matrix adhesion ECL imaging were performed by using confocal laser scanning microscopy (CLSM) on a Leica TCS SP8 STED microscope (Leica Microsystems Inc., Exton, PA) and a potentiostat (CHI 660D electrochemical workstation). A HyD detector and 100× oil-immersion objective (NA 1.4) were used for ECL imaging. A 775-nm donut beam was used to improve the spatial resolution. The ECL measurements were carried out on MPI-E detection system (Xi’an Remex, China) with a 3-electrode system. The image analysis and processing were performed by Matlab and ImageJ softwares.

## Data Availability

Data supporting the findings of this study are available in the main text or the Supplementary Materials. Additional data related to this paper may be requested from the authors.
