# Supplementary material for: Reversibly Tuning Electrochemiluminescence with Stimulated Emission Route for Single-Cell Imaging
Source: Research (Wash D C). 2023 Oct 18;6:0257. doi: 10.34133/research.0257 (PMC11776023; doi:10.34133/research.0257)
Supplement: Supplementary 1 — Figs. S1 to S22 Movies S1 and S2 [file research.0257.f1.zip › Supporting Information.pdf]

Supporting Information

Reversibly Tuning Electrochemiluminescence with Stimulated Emission Route for Single Cell Imaging

Cheng Ma, Xiaodan Gou, Zejing Xing, Min-Xuan Wang, Wenlei Zhu, Qin Xu, Dechen Jiang* and Jun-Jie Zhu*

Abstract: Electrochemiluminescence (ECL) has established itself as an excellent transduction technique in biosensing and light-emitting device, while conventional ECL mechanism depending on spontaneous emission of luminophores lack of reversibility and tunable emission characters, limiting the universality of ECL technique in the fields of fundamental research and clinical applications. Here, we report the first observation of stimulated emission route in ECL and thus establish a reversible tuning ECL microscopy for single cell imaging. This microscopy uses a focused red-shifted beam to transfer spontaneous ECL into stimulated ECL, which enables selective and reversible tuning of ECL emission from homogeneous solution, single particles and single cells. After excluding other possible competitive routes, the stimulated ECL emission route is confirmed by a dual-objective system in which the suppressed spontaneous ECL is accompanied by the enhanced stimulated ECL. By incorporating a commercial donut-shaped beam, the sharpness of single cell matrix adhesion is significantly improved 2-3 times compared with the counterpart in confocal ECL mode. The successful establishment of this stimulated emission ECL will greatly advance the development of light-emitting device and super-resolution ECL microscopy.

Table of Contents

Table of Contents 2
Results and Discussion 3
Description of Movies 12
References 12

Results and Discussion

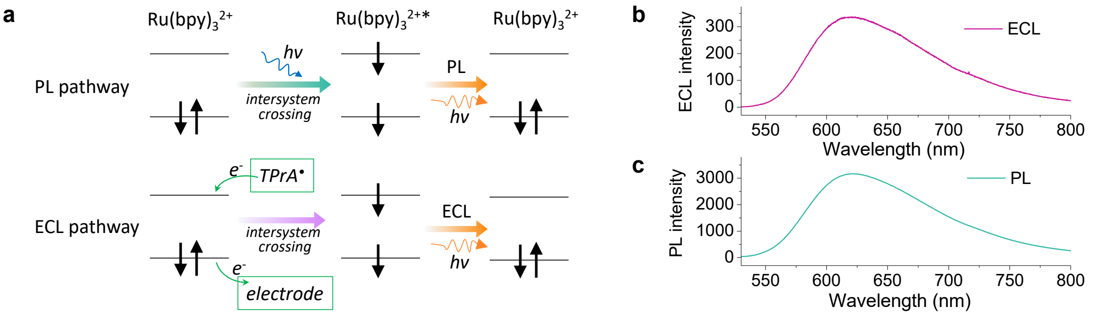

Figure S1. (a) Schematic diagrams showing the principles of PL and ECL pathways of Ru(bpy)$_3^{2+}$. In PL pathway, the incident photons with a high energy trigger the metal to ligand (d-π*) charge transfer (MLCT) transition from the ground state (S_0) to the singlet excited state (S_1). Rapid intersystem crossing and vibration relaxation to the lowest triplet states (T_1) of Ru(bpy)$_3^{2+}$ eventually result in the observed photon emission at approximately 620 nm. Similarly, the ECL pathway of Ru(bpy)$_3^{2+}$ obeys the same emission processes except that the formation of the singlet excited state (S_1) is caused by highly energetic electron-transfer reactions. (b,c) ECL and PL spectra of Ru(bpy)$_3^{2+}$. Because of the same radiative transition mechanism, identical emission spectra with the wavelength of maximum emission at 620 nm are observed in both PL and ECL spectra.

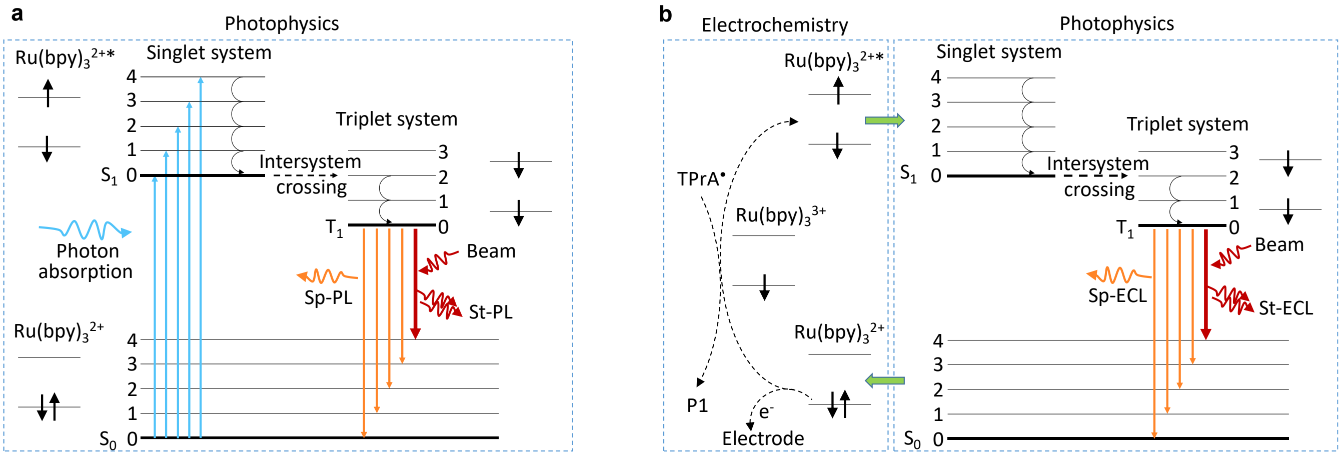

Figure S2. Jablonski diagram indicating the stimulated emission pathways of PL (a) and ECL (b) under 730 nm beam irradiation.

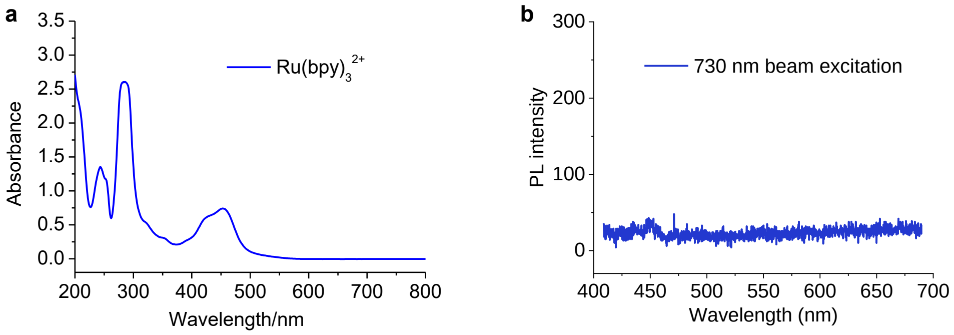

Figure S3. (a) Absorption spectrum of Ru(bpy)$_3^{2+}$ showing negligible absorbance at 730 nm. (b) PL spectrum of Ru(bpy)$_3^{2+}$ indicating no PL emission under the 730 nm beam excitation.

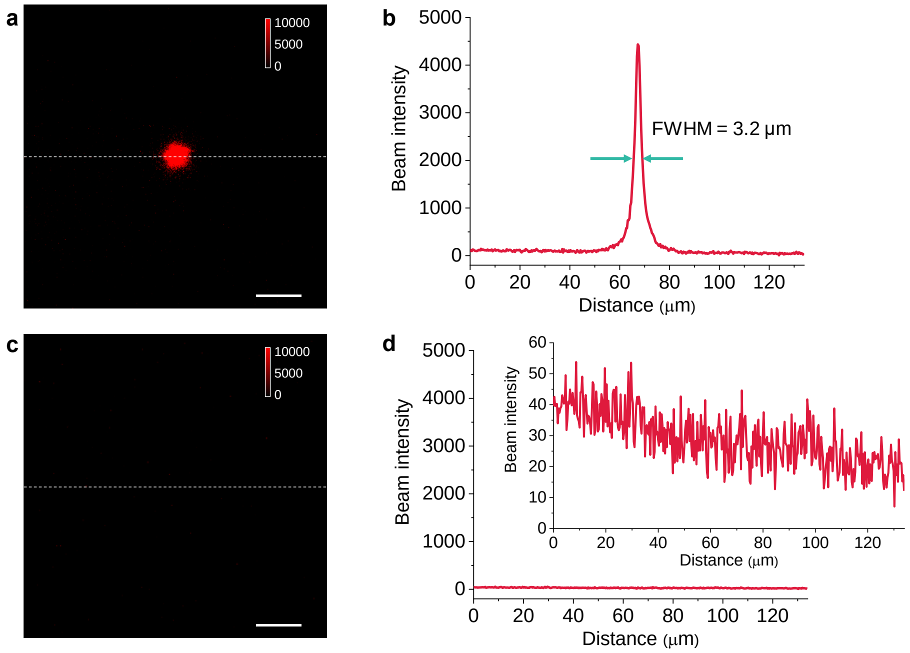

Figure S4. (a) 730 nm beam spot on ITO surface after focused by the objective. (b) Beam intensity lateral profile across the dash line in (a) showing that the point-spread-function (PSF) of the focused beam featured a full-width-at-half-maximum (FWHM) of 3.2 μm in the lateral direction. The focused beam power is 48.8 μW/μm^2. (c) The same region as in (a) after a premium bandpass filter (center wavelength 620 nm, FWHM 52 nm) is placed in front of the camera so that the reflected 730 nm beam is blocked by the filter. (d) Beam intensity profile across the dash line in (c) showing that negligible back-propagating 730 nm beam is collected in camera. Inset shows a zoom-in view of the ECL intensity across the dash line. Scale bar (white) is 20 μm.

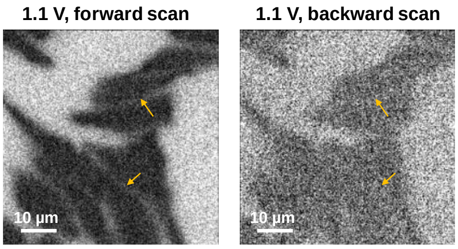

Figure S5. ECL snapshots of attached HeLa cells on ITO electrode. The left and right panels denote the ECL image of HeLa cells at 1.1 V in the forward and backward scan, respectively, during a potential cycle (0.8 V to 1.4 V) at a scan rate of 0.1 V/s. The electrolyte is 200 mM PBS (pH 7.0) containing 200 μM $Ru(bpy)_3^{2+}$ and 20 mM TPrA. The yellow arrows indicate cell-cell junctions. Scale bar (white) is 10 μm.

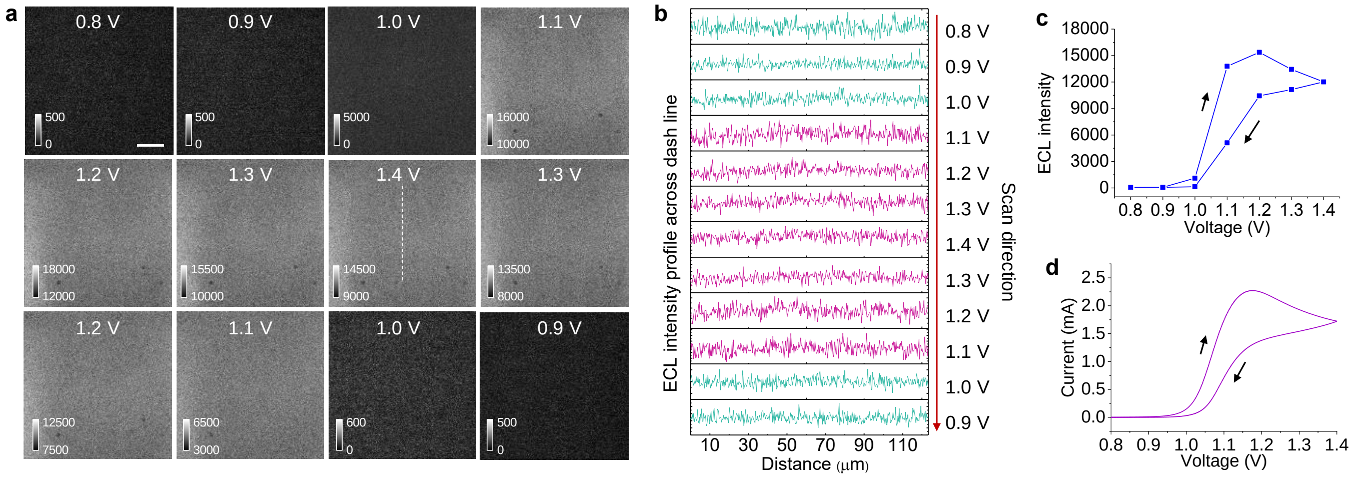

Figure S6. (a) ECL snapshots without beam irradiation during a potential cycle (0.8 V to 1.4 V) at a sweep rate of 0.1 V/s in 200 mM PBS (pH 7.0) containing 200 μM $Ru(bpy)_3^{2+}$ and 20 mM TPrA. Exposure time is 200 ms. Scale bar (white) is 30 μm. (b) ECL intensity profiles across the dash line in (a) during the potential cycle sweep. (c) ECL intensity in (a) as a function of electrode potential. (d) The corresponding cyclic voltammetry curve.

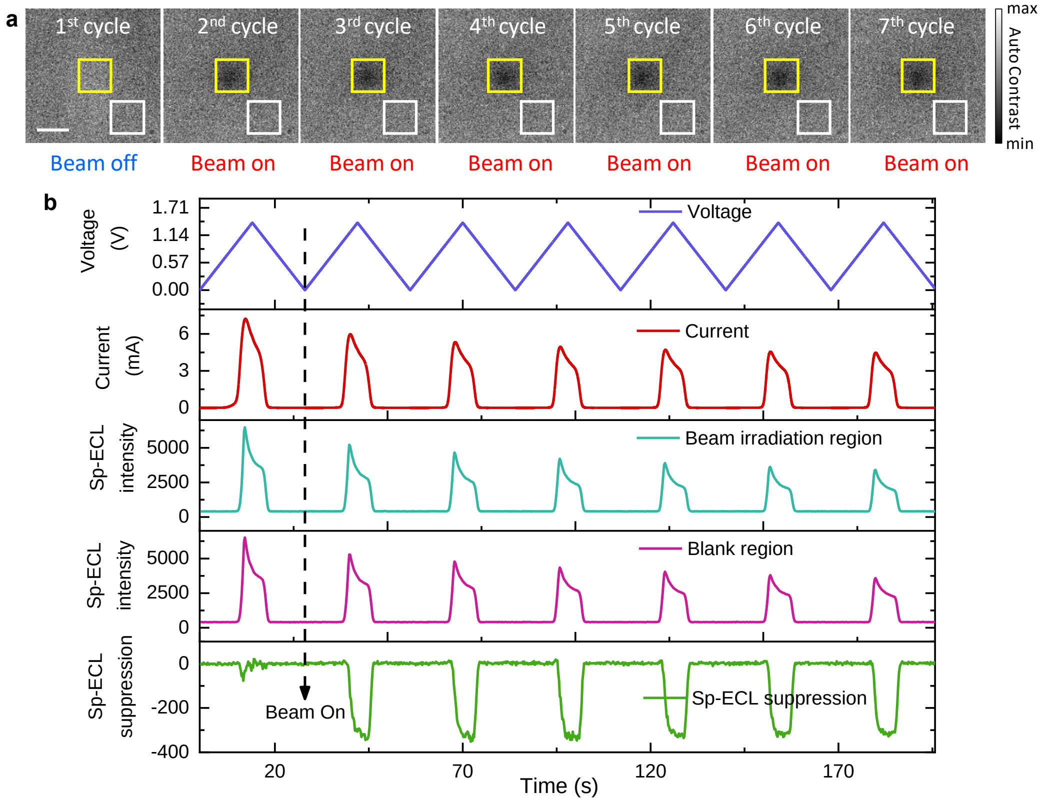

Figure S7. (a) ECL snapshots during seven consecutive CV scans. The yellow and white boxes indicate the beam irradiation and blank regions, respectively. In the 1st cycle, the beam turns off. But then the beam turns on from the 2nd cycle to 7th cycle. The potential is cyclically scanned from 0 to 1.4 V at a scan rate of 0.1 V/s in 200 mM PBS (pH 7.0) containing 200 μM $Ru(bpy)_3^{2+}$ and 20 mM TPrA. Exposure time is 200 ms. Scale bar (white) is 30 μm. (b) Under seven consecutive CV scans, the profiles of the current (red line), Sp-ECL intensity in beam irradiation region (cyan line) and in blank region (purple line) with time elapsing. The Sp-ECL suppression curve (green line) is obtained by subtracting Sp-ECL intensity in blank region (purple line) from that in beam irradiation region (cyan line).

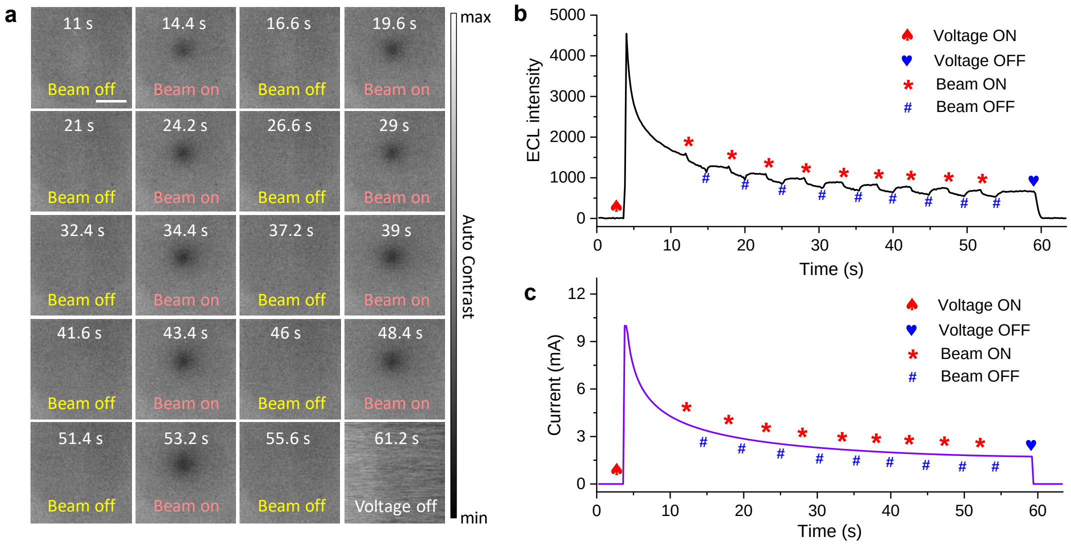

Figure S8. (a) Under a constant 1.3 V potential, ECL snapshots with time elapsing when the beam is successively switched. Exposure time is 200 ms. Scale bar (white) is 50 μm. Electrolyte is 200 mM PBS (pH 7.0) containing 2 mM $Ru(bpy)_3^{2+}$ and 25 mM TPrA. ECL intensity trajectory in the beam irradiation region (b) and the corresponding current trajectory (c) when the beam successively turns on and off.

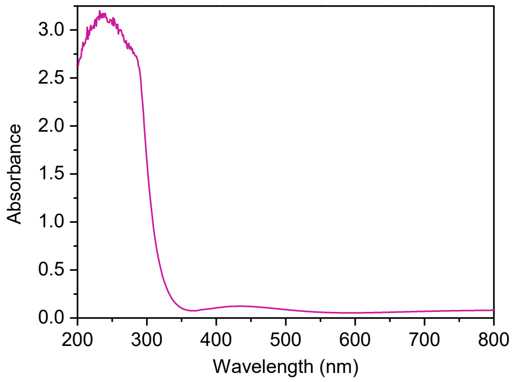

Figure S9. Absorption spectrum of ITO electrode.

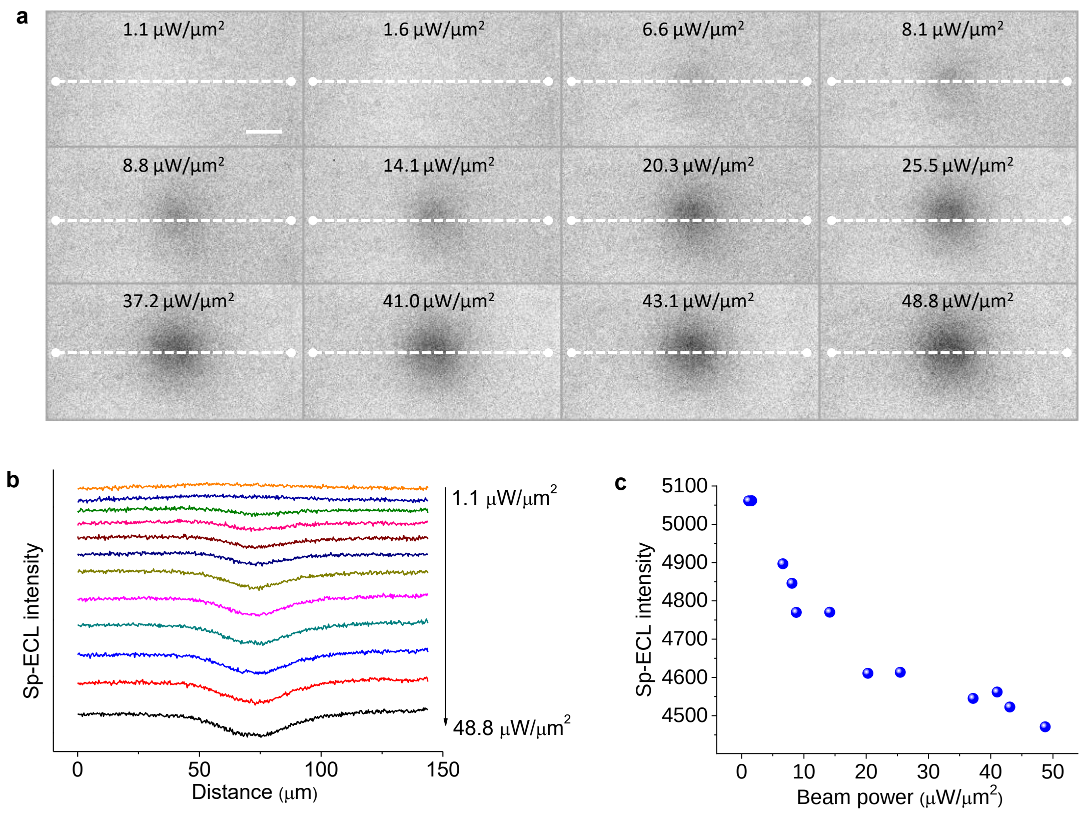

Figure S10. (a) Sp-ECL images under beam irradiation with different powers. Scale bar (white) is 20 μm. Electrolyte is 200 mM PBS (pH 7.0) containing 1 mM $Ru(bpy)_3^{2+}$ and 50 mM TPrA. Constant potential: 1.3 V. (b) Sp-ECL intensity profiles across the dash line in (a) under beam irradiation with different powers. (c) Sp-ECL intensity in beam irradiation region as a function of beam power, showing the quenching degree of Sp-ECL by increasing beam power.

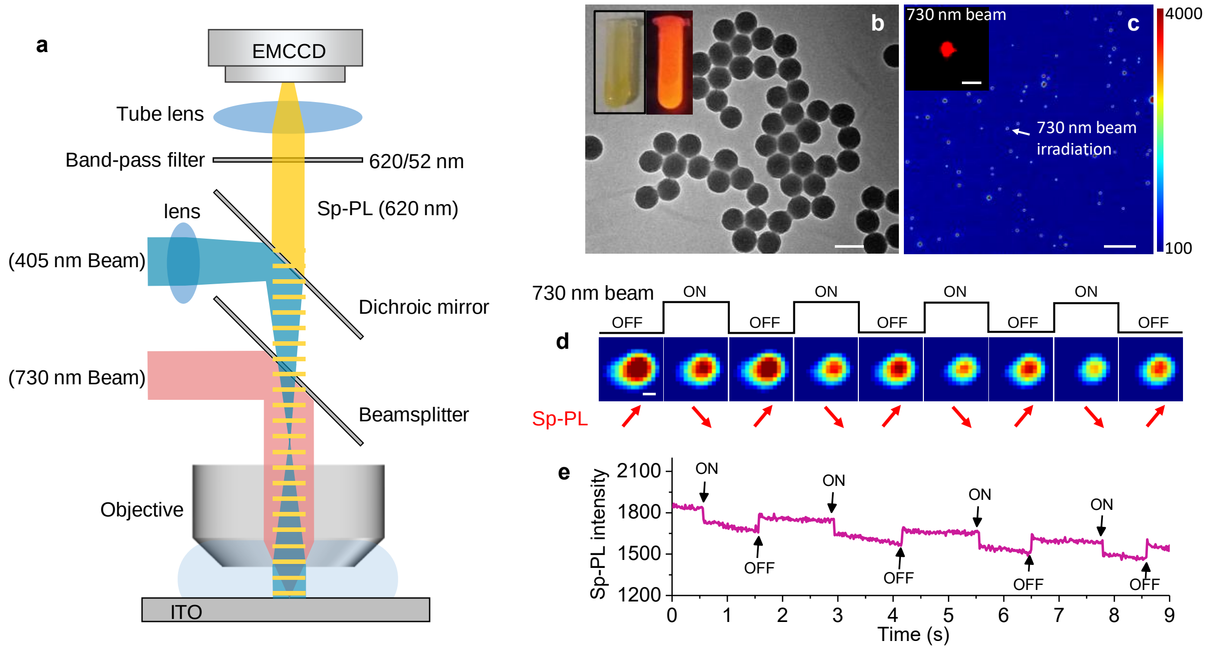

Figure S11. (a) Schematic illustration of PL microscopy setup with a 405 nm excitation beam and a 730 nm beam. The 405 nm beam as the excitation light of $Ru(bpy)_3^{2+}$ is focused on the back focus plane of objective and forms a collimated excitation light for wide-field PL imaging. The collimating 730 nm beam floods into the back focal plane of objective and creates a focused spot to induce stimulated emission PL (St-PL). A band-pass filter (620/52 nm) in front of EMCCD blocks the back-propagating 730 nm beam and 405 nm excitation beam. Only spontaneous PL (Sp-PL) is allowed to pass through the band-pass filter and collected by EMCCD. (b) TEM image of $Ru(bpy)_3^{2+}$-doped silica nanoparticles (RuDSNs). Scale bar (white) is 100 nm. Inset: the photograph of RuDSNs solution (left) and the corresponding PL photograph (right) under UV light excitation. (c) PL image of single RuDSNs on ITO surface when excited by 405 nm light. The arrow indicates the single RuDSN also irradiated by focused 730 nm beam. Scale bar (white) is 20 μm. Inset: the image of 730 nm beam spot after removing the band-pass filter (620/52 nm). Scale bar (white) is 5 μm. (d) PL image sequence of the single RuDSN with continuously switching 730 nm beam ON and OFF. Scale bar (white) is 1 μm. (e) Sp-PL intensity trajectory of the single RuDSN in (d) with continuously switching 730 nm beam ON and OFF.

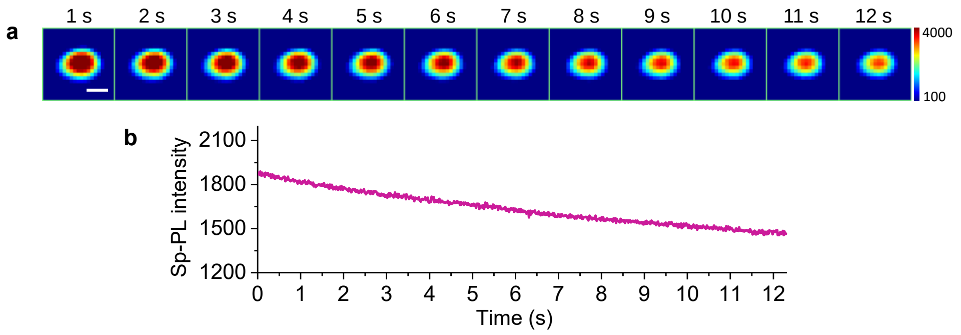

Figure S12. (a) PL image sequence of single RuDSN without 730 nm beam irradiation. Scale bar (white) is 2 μm. (b) Sp-PL intensity trajectory of the single RuDSN in (a) with time elapsing.

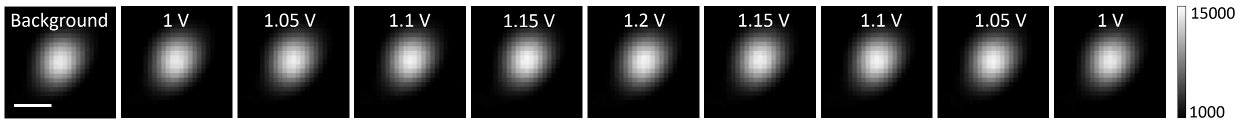

Figure S13. The sum of the incident 730 nm beam and the transmitted St-ECL from inverted microscope. Scale bar (white) is 5 μm. Scan rate is 0.1 V/s. Electrolyte is 200 mM PBS (pH 7.0) containing 400 μM $Ru(bpy)_3^{2+}$ and 30 mM TPrA. Exposure time of CCD are 100 ms.

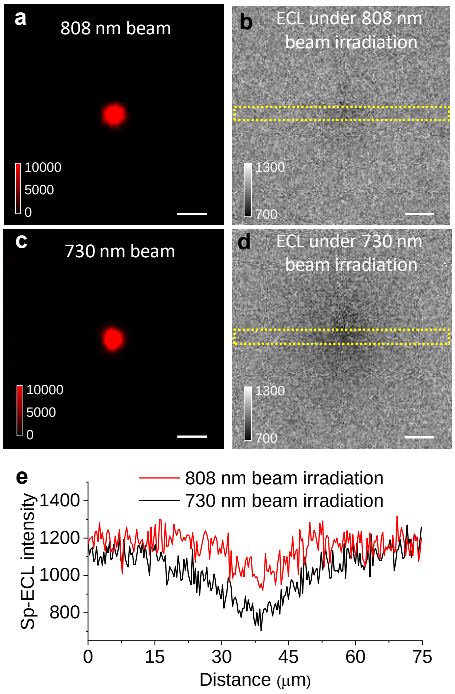

Figure S14. (a) 808 nm beam spot after removing the band-pass filter (620/52 nm). (b) ECL image under 808 nm beam irradiation. (c) 730 nm beam spot after removing the band-pass filter (620/52 nm). (d) ECL image under 730 nm beam irradiation. (e) Lateral ECL intensity profiles across the yellow box in (b,d). The power of 808 nm and 730 nm beams are both 8.8 μW/μm^2. Electrolyte is 200 mM PBS (pH 7.0) containing 500 μM $Ru(bpy)_3^{2+}$ and 50 mM TPrA. Voltage: a constant 1.3 V. Scale bar (white) is 10 μm.

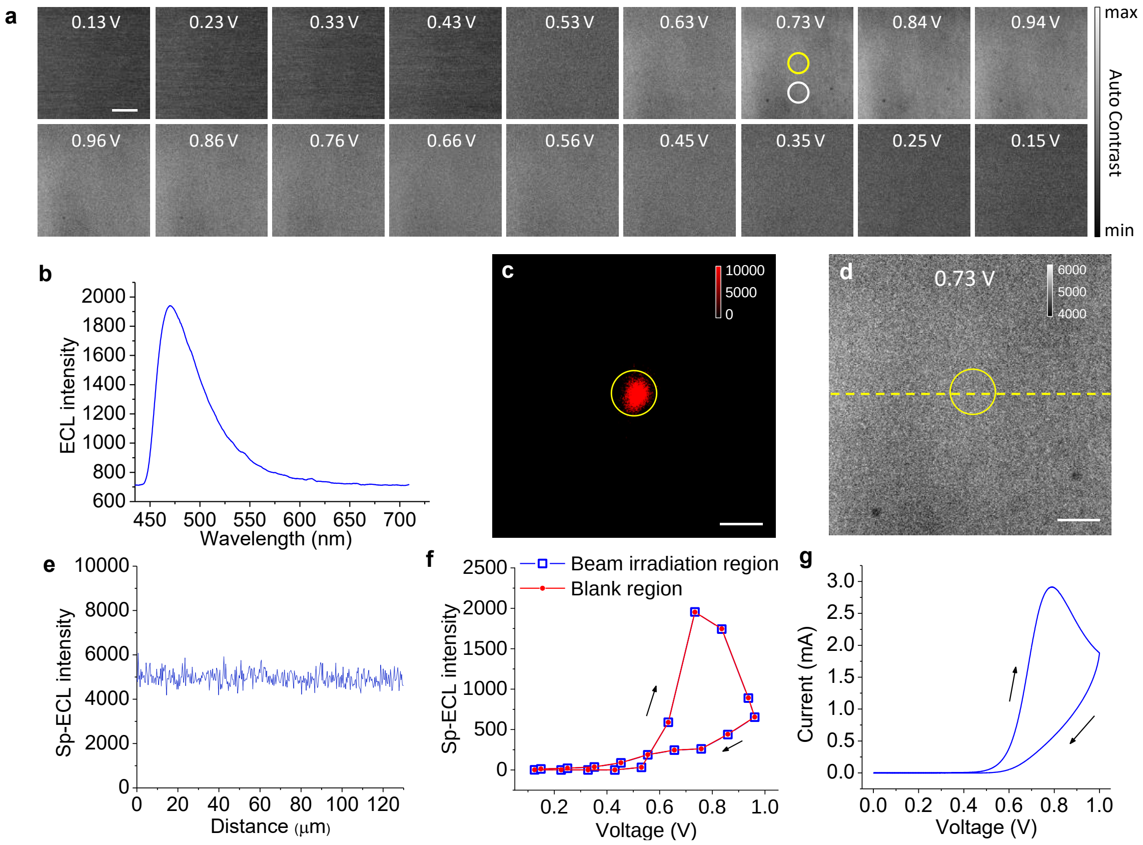

Figure S15. (a) ECL image sequence under 730 nm beam irradiation during a potential cycle (0 V to 1 V) at a scan rate of 0.1 V/s in 200 mM PBS (pH 7.0) containing 200 μM L012 and 1 mM H_2O_2. The yellow and white circles represent the irradiation and blank regions, respectively. The back-propagating 730 nm light is blocked by a band-pass filter (460/60 nm) in front of camera. Exposure time is 500 ms. Scale bar (white) is 30 μm. (b) ECL spectrum of L012. (c) 730 nm beam spot in the yellow circle after removing the band-pass filter (460/60 nm). (d) ECL image at 0.73 V showing a uniform ECL intensity distribution under 730 nm beam irradiation in the yellow circle. Scale bar (white) is 20 μm. (e) Sp-ECL intensity profiles across the dash line in (d). (f) Sp-ECL intensity in beam irradiation (yellow circle) and blank (white circle) regions in (a) as a function of electrode potential. (g) The corresponding cyclic voltammetry curve.

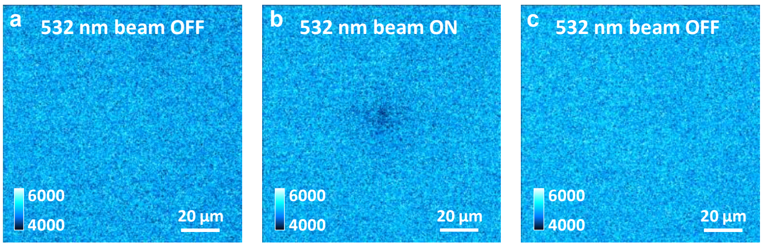

Figure S16. Under a constant 0.8 V potential, ECL snapshots (a) and ECL snapshots when the beam is switched on (b) and then switched off (c). Exposure time is 500 ms. Scale bar (white) is 20 μm. Electrolyte is 200 mM PBS (pH 7.0) containing 200 μM L012 and 1 mM H_2O_2.

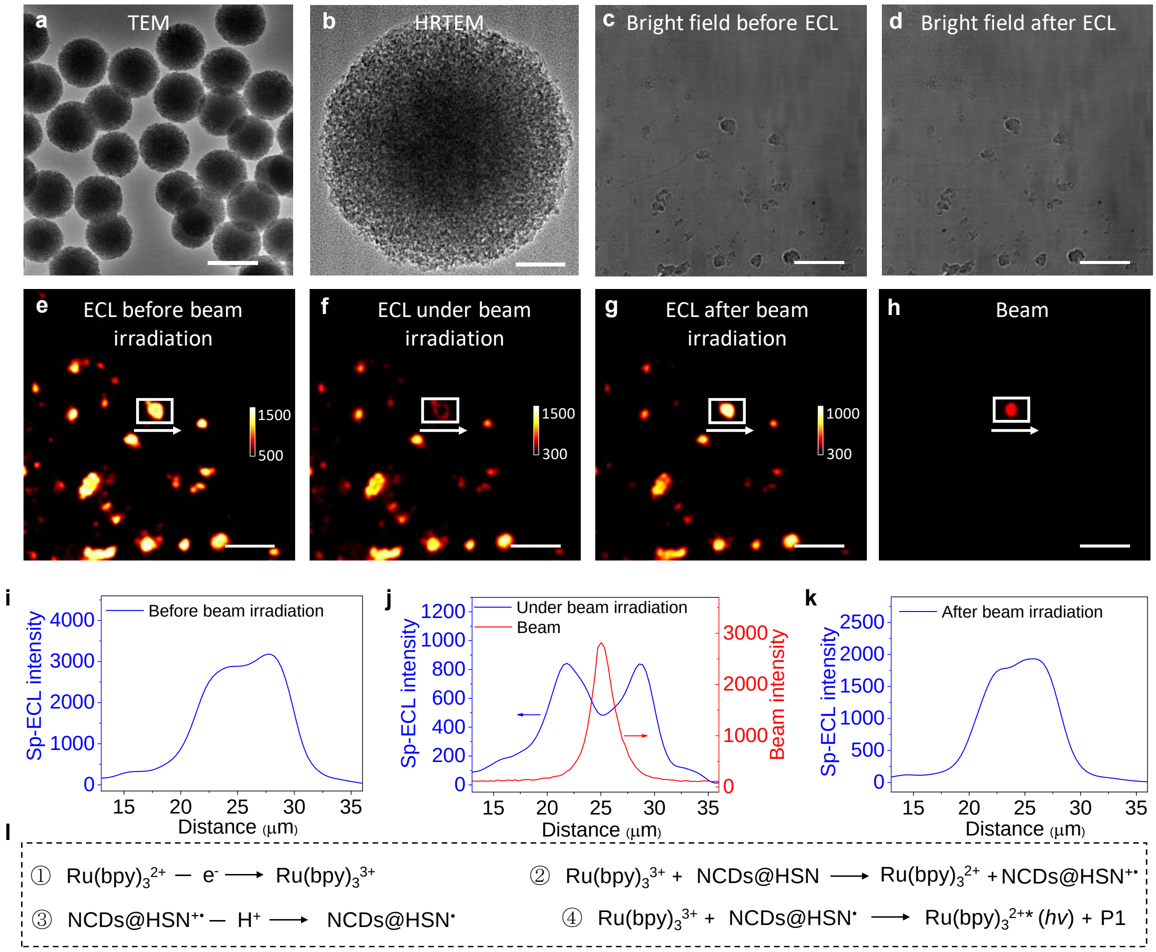

Figure S17. (a) TEM image of NCDs@HSN. Scale bar (white) is 300 nm. (b) High resolution TEM image of NCDs@HSN. Scale bar (white) is 50 nm. (c,d) Bright field images of NCDs@HSN on ITO electrode before and after ECL reactions. (e,f,g) ECL images of NCDs@HSN before, under and after 730 nm beam irradiation. Constant voltage: 1.3 V. Electrolyte is 200 mM PBS (pH 7.0) containing 2 mM $Ru(bpy)_3^{2+}$. (h) 730 nm beam spot after removing the band-pass filter (620/52 nm). Scale bar (white) is 30 μm. (i) Sp-ECL intensity profile across the white box in (e) along the arrow direction. (j) Sp-ECL intensity profile across the white box in (f) along the arrow direction, which is overlaid with the beam intensity profile across the white box in (h). (k) Sp-ECL intensity profile across the white box in (g) along the arrow direction. (l) Catalytic route ECL mechanism by electron transfer reactions between freely diffusing $Ru(bpy)_3^{2+}$ and NCDs@HSN under anodic potential.

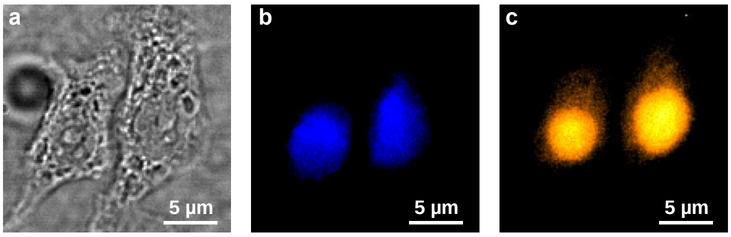

Figure S18. (a) Bright field image of HeLa cells on ITO electrode. (b) Fluorescence image of nuclear staining with DAPI. (c) The corresponding ECL image. Cells are fixed with paraformaldehyde for 30 min and pretreated with 0.1% Triton X-100 for 10 min. Voltage: a constant 1.3 V (vs. Ag/AgCl). Electrolyte: 200 mM PBS (pH 7.0) containing 2 mM $Ru(bpy)_3^{2+}$. Scale bar (white) is 5 μm.

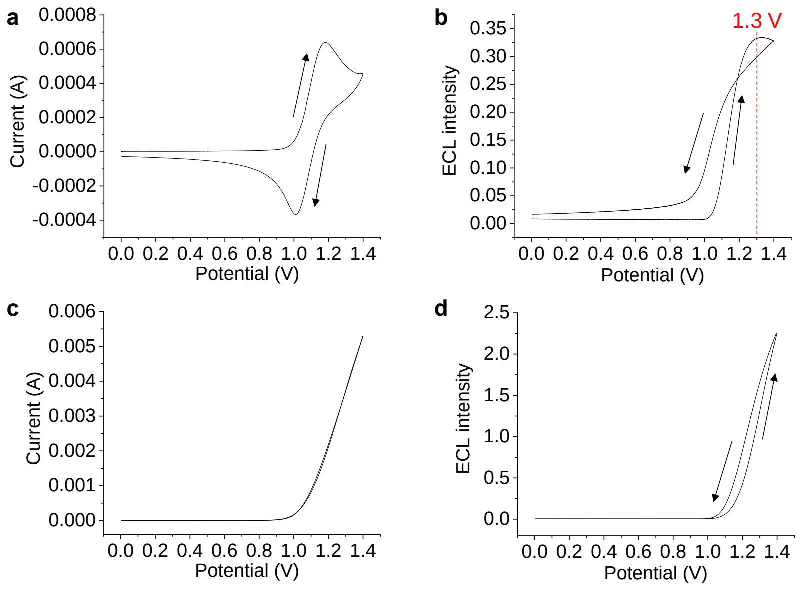

Figure S19. (a) CV curves and (b) ECL responses for 2 mM $Ru(bpy)_3^{2+}$ in 200 mM PBS (pH 7.4). The voltage of PMT is set to 350 V. (c) CV curves and (d) ECL responses for 1 mM $Ru(bpy)_3^{2+}$ and 100 mM TPrA in 200 mM PBS (pH 7.4). The voltage of PMT is set to 150 V.

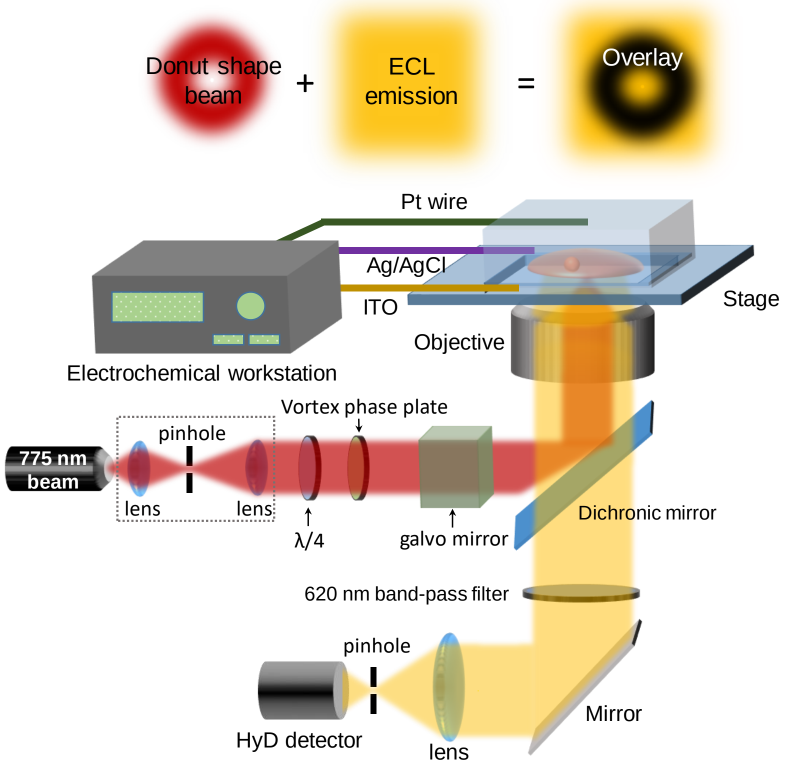

Figure S20. Schematic illustration of stimulated emission depletion ECL imaging setup. In this setup, an auxiliary 4f-system consisted of two lens and one pinhole, a 4/λ retarder, a vortex phase plate was used to generate the doughnut-shaped intensity distribution in the focus of the STED beam. A galvo mirror system was used to make the donut-shaped beam scanning the sample. An electrochemical workstation with the three electrodes system (ITO as work electrode, Ag/AgCl as reference electrode, Pt wire as counter electrode) was used to offer a potential that generated ECL emission on ITO work electrode. A dichronic mirror and other filters were used to prevent the back-propagating 775 nm beam from entering the HyD detector. Therefore, only spontaneous ECL in the center of donut-shaped beam can be recorded.

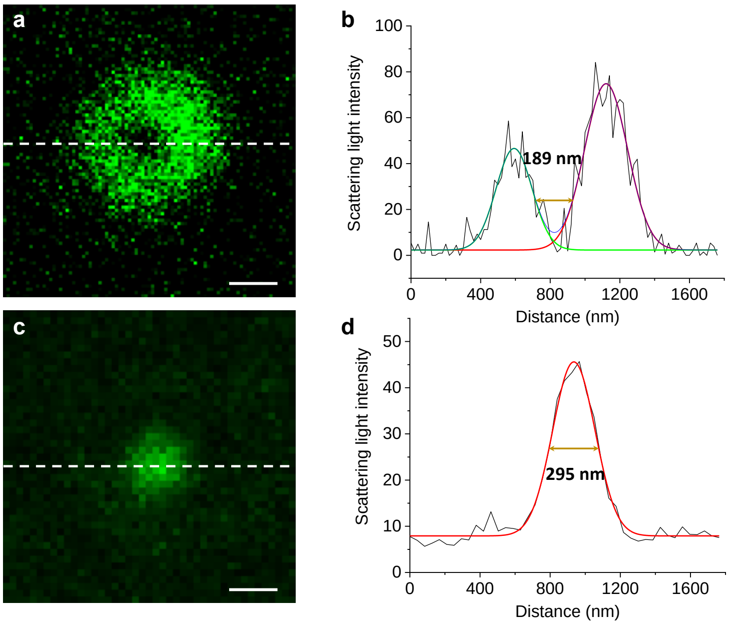

Figure S21. (a) Donut-shaped beam pattern. The donut-shaped pattern is recorded by scanning the STED beam laterally over a scattering 100 nm gold nanoparticle. Scale bar (white) is 300 nm. (b) Donut-shaped beam pattern intensity profile across the dash line in (a). The black line is the original intensity profile. The red and green lines are the two fitting peak curves with Gaussian equation. The blue line is the overall fitting two peak curves. The FWHM is 189 nm at the valley. (c) Excitation beam pattern. The excitation beam pattern is recorded by scanning the excitation beam laterally over a scattering 100 nm gold nanoparticle. Scale bar (white) is 300 nm. (d) Excitation beam pattern intensity profile across the dash line in (c). The black line is the original intensity profile. The red line is the fitting peak curve with Gaussian equation. The FWHM is 295 nm for the peak.

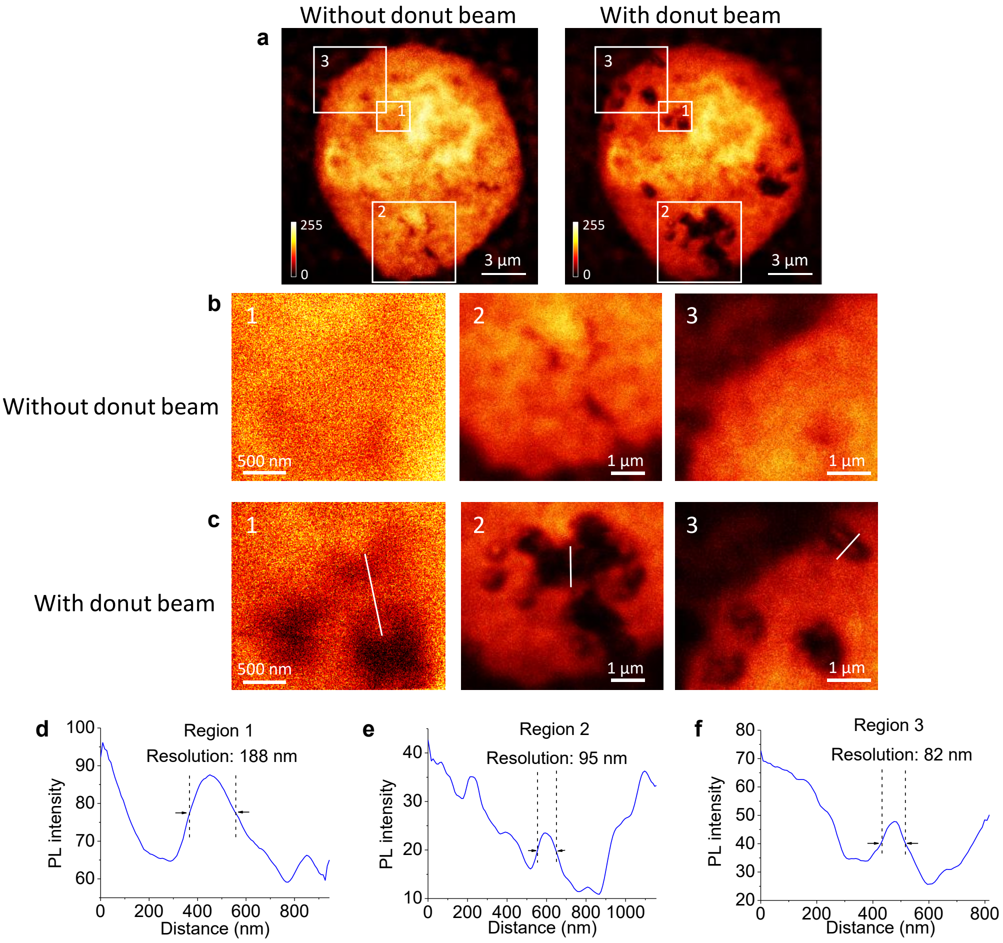

Figure S22. (a) Confocal PL images of single cell nucleus stained with $Ru(bpy)_3^{2+}$ in the absence and presence of donut beam. Cells are fixed with paraformaldehyde for 30 min. The excitation wavelength is 470 nm and the emission is collected between 550 nm and 680 nm. (b,c) Three magnified regions of interest marked with 1,2,3 in (a) for comparison. (d,e,f) PL intensity profiles across the white lines in (c) showing the improved spatial resolution.

Description of Movies

Movie S1. Sp-ECL at the irradiation region by switching on 730 nm beam during successive cyclic voltammetry scanning. (The potential is cyclically scanned from 0 to 1.4 V at a scan rate of 0.1 V/s in 200 mM PBS (pH 7.0) containing 200 μM $Ru(bpy)_3^{2+}$ and 20 mM TPrA. The focused beam power is 8.1 μW/μm^2. Exposure time is 200 ms.)

Movie S2. Reversibility of suppressed Sp-ECL induced by 730 nm beam which is successively switched on and off. (Constant potential: 1.3 V vs. Ag/AgCl reference electrode. Exposure time: 200 ms. The focused beam power is 20.3 μW/μm^2. Electrolyte is 200 mM PBS (pH 7.0) containing 2 mM $Ru(bpy)_3^{2+}$ and 25 mM TPrA.)

References

[1] I. Rubinstein, A. J. Bard, *J. Am. Chem. Soc.* **1980**, *102*, 6641-6642.

[2] L. H. Zhang, S. J. Dong, *Anal. Chem.* **2006**, *78*, 5119-5123.

[3] a) J. Sun, J. Zhang, M. Zhang, M. Antonietti, X. Fu, X. Wang, *Nature communications* **2012**, 1139; b) C. Ma, H.-F. Wei, M.-X. Wang, S. Wu, Y.-C. Chang, J. Zhang, L.-P. Jiang, W. Zhu, Z. Chen, Y. Lin, *Nano. Lett.* **2020**, *20*, 5008-5016.

[4] a) B. Han, Y. Li, T. Peng, M. Yu, X. Hu, G. He, *Analytical Methods* **2018**, *10*, 2989-2993; b) C. Ma, M. X. Wang, H. F. Wei, S. Wu, J. R. Zhang, J. J. Zhu, Z. Chen, *Chem. Commun.* **2021**, *57*, 2168-2171.

[5] C. Ma, W. Wu, L. Li, S. Wu, J. Zhang, Z. Chen, J.-J. Zhu, *Chem. Sci.* **2018**, *9*, 6167-6175.

[6] C. Ma, W. Wu, Y. Peng, M. X. Wang, G. Chen, Z. Chen, J. J. Zhu, *Anal. Chem.* **2018**, *90*, 1334-1339.
